# Characterization of Friction Stir and TIG Welded CK45 Carbon Steel

**DOI:** 10.3390/ma14154098

**Published:** 2021-07-23

**Authors:** Mohammadreza Rafati, Amir Mostafapour, Hossein Laieghi, Mahesh Chandra Somani, Jukka Kömi

**Affiliations:** 1 Faculty of Mechanical Engineering, University of Tabriz, PO Box 51666-16471 Tabriz, Iran; Eng.rafmr@gmail.com (M.R.); Hossein.laieghi@gmail.com (H.L.); 2 Centre for Advanced Steels Research, University of Oulu, PO Box 4200, 90014 Oulu, Finland; mahesh.somani@oulu.fi (M.C.S.); jukka.komi@oulu.fi (J.K.)

**Keywords:** carbon steel, macrostructure, microstructure, mechanical properties, friction stir welding (FSW), tungsten inert gas (TIG) welding

## Abstract

The present paper aims to compare the microstructural and mechanical properties of CK45 carbon steel plates, joined by friction stir (FSW) and tungsten inert gas (TIG) welding methods. Besides visual inspection, the welded joints and the base material were subsequently evaluated in respect of optical microstructures, hardness and tensile properties. Sound joints could be accomplished using both the FSW and TIG welding methods through proper selection of process parameters and the filler metal. The influence of a water-cooling system on the FSW and various filler metals on the quality of TIG welding were further assessed. Both the FS welded sample as well as TIG welded samples with two different filler metals ER70S-6 and ER80S-B2 exhibited brittle behavior that could be mitigated through optimized water cooling and use of R60 filler metal. A drastic reduction of brittle martensite phase constituent in the microstructure corroborated significant improvements in mechanical properties of the welded zones for both the FSW sample as well as TIG welded samples with R60 filler metal.

## 1. Introduction

Carbon steels are extensively used in both consumer as well as commercial applications. Joining them via welding methods, however, may encounter severe problems including the formation of brittle phases like martensite, hydrogen-induced cracks and solidification cracks, thus leading to the failure of components prematurely. Among the conventional fusion welding methods, tungsten inert gas (TIG) welding (also known as GTAW, i.e., gas tungsten arc welding) with superb weld quality and precise control is extensively utilized in industrial applications. In this fusion welding process, an electric arc is generated between a non-consumable tungsten electrode and the workpiece to create an intense thermal energy, which melts the metals to be joined. Both the weld area and the electrode are protected from oxidation or any atmospheric contamination by shielding with an inert gas. In comparison with gas metal arc welding (GMAW), TIG welding is confined to weld thicker materials (more than 3 mm) in a single pass due to its shallow penetration [1,2,3]. The usual technique to solve this problem is by resorting to a single-V or double-V bevel shape weld type. Also, the selection of a proper set of welding parameters and filler metals plays the key role in promoting grain refinement and improvement of mechanical properties [4,5]. The selection of filler metals is highly dependent on the phase structure, chemical composition and mechanical properties of the joints. The alloying elements of filler metals can significantly affect the weld bead configuration as well as the formation of new grain boundary structures and phases resulting in significant impact on the mechanical properties and defect formation. Compared to the TIG welding method, friction stir welding (FSW) has remarkable benefits with respect to lower heat input, joint quality, and better mechanical properties [6]. This process is renowned as a solid-state joining technology in which intense frictional heat is generated via a non-consumable tool rotating along the intersection of two clamped workpieces [7,8]. Considering the nature of this method, the temperature is maintained under the melting point. Consequently, the possibility of gas porosities in the weld region as a result of trapped gas in the molten weld pool is completely eliminated [9]. With no gas porosity formation in the weld region as well as no need for any filler materials, the overall cost of welding by FSW can find substantial reduction, unlike in the case of TIG welding. In accord with its merits, the FSW process has found wide applications in the aeronautical industries, where the joining of lightweight alloys is a crucial issue. In recent years, this process has attracted enormous attention of researchers to use the technique for joining carbon steels in various applications [10]. However, the FSW of carbon steels or high-temperature materials is invariably facing problems, such as proper tool material selection, in which case the intactness of weld joints at high temperatures is vital [11].

A series of studies have been carried out on developing the FSW process for carbon steels, thus imparting grain refinement at the stir zone with a consequent improvement in mechanical properties. For example, Sato et al. [12] employed polycrystalline cubic boron nitride (PCBN) tool to join ultrahigh carbon steel during the FSW, thereby leading to the realization of defect-free welds. There were no significant differences in the hardness and microstructure of all the welds. Microstructural observation exhibited the alteration of duplex structure (ferrite + cementite) into the martensitic structure at the center of the weld line, while the heat affected zone (HAZ) consisted of a mixed microstructure comprising both the martensitic as well as duplex (ferrite + cementite) phase constituents. Cui et al. [13] carried out an investigation on the FSW of a high-carbon steel, S70C (0.72 wt.% C) under two different conditions of rotational and welding speeds. They exhibited a significant dependence of microstructure and mechanical properties on the welding parameters, in particular to the cooling rate and peak temperature. Thus, the FSW of high-carbon steels enables realization of defect-free joints by controlling the temperature. As stated by Fujii et al. [14], both the microstructures and mechanical properties of ultrafine-grained interstitial free steels (20 ppm-C), processed via accumulative roll-bonding (ARB), remained unchanged during the FSW because of the extra low carbon content. Mild steels contain 0.05–0.25% carbon, which can be rendered ductile and formable via suitable heat treatments. A feasibility study of FSW of hot-rolled 1018 mild steel sheets (6.3 mm thick) was conducted by Lienert et al. [15] at a traverse speed ranging from 25 to 100 mm/min, although the results were reported only for 25 mm/min traverse speed. The tool materials selected for FSW were tungsten-based and molybdenum-based alloys with the maximum temperature of the weld zone surpassing 1200 °C. Mechanical tests indicated relatively greater tensile strength for HAZ compared to the base material, thus resulting in fractures in the base material during tensile testing.

Medium-carbon steels are utilized for fabricating shafts, gears, axles, crankshafts, forgings, and couplings. Steels, possessing carbon from 0.4% to 0.6%, have been found to be suitable for applications in rails, rail axles and, railway wheels [16]. Considering their wide range of applications, it is inevitably important to have a deep understanding of the behavior of these steels during the welding process. Among the medium-carbon steels, the CK45 carbon steel is broadly used in different industries, such as those dealing in car and engine parts as well as industrial shafts and rollers. Additionally, CK45 carbon steel is regularly applied in making the pump shafts owing to its low cost. However, only limited research has thus far been conducted on the joining of CK45. The primary objective of the present research is to conduct a feasibility study of the FSW process on CK45 carbon steel and compare the process in respect of microstructures and properties obtained via TIG welding. The results of both FSW and TIG welding processes were assessed to obtain a comprehensive understanding of the joint properties.

## 2. Materials and Methods

**Figure 1:**
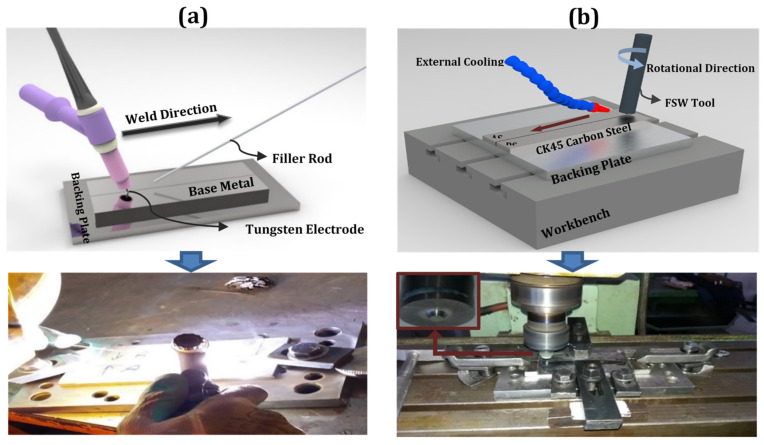
Schematic representation of weld arrangement and actual pictures of (**a**) tungsten inert gas (TIG) and (**b**) friction stir welding (FSW).

The nominal chemical compositions of the CK45 carbon steel plates and filler metals used in the study are shown in Table 1. Rolled plates of the steel with dimensions of 160 mm length, 26 mm width and 5 mm thick were prepared in order to join via fusion welding (using TIG technique) and FSW. Prior to this, all samples were subjected to normalizing treatment by reheating at 920 °C for 15 min, followed by cooling in air. To accomplish the fusion welds, three different fillers (ER70S-6, ER80S-B2, and R60) were utilized in the course of TIG welding, whose details along with other process parameters are listed in Table 2. Comprising of deoxidisers, the ER70S-6 filler metal provides better material yield and enhanced capacity for faster welding travel speed resulting in high productivity and increased life of consumables. ER70S-6, filler metal, imparts relatively higher strength owing to elimination of the pores and ferrite structure in the weld zone. The second filler metal selected for the present study was ER80S-B2. This filler metal provided the advantage of a strength match in the weld deposit without inducing crack sensitivity. It was considered interesting to compare the result with that of ER70S-6 filler metal, especially the effect of Mo addition. Another type of filler metal, R60 was also used, which was originally designed to provide porosity-free welds, resulting in high tensile strength. Owing to the significant Mn-level (0.93 wt.%) in this filler, the crack susceptibility can be markedly reduced, and can lead to a positive impact on mechanical properties. In general, a high level of Mn and Si in the filler increases the fluidity of the weld pool, thus creating a smooth appearance and resulting in minimal post-weld grinding. A schematic of the weld process and the actual picture of the TIG welding operation are shown in Figure 1. The compositions of the fillers have resembled the base metal (BM). The CK45 plates were subjected to preheating up to 100 °C, thereby marginally bringing down the thermal gradient between the filler and the BM. However, preheating the plates practically contributed to tackling the thermal shocks.

For the FSW process, a tungsten carbide tool consisting of a convex shoulder with a diameter of 16 mm and a 5 mm-in-upper-diameter conical pin was employed. In the first step of welding, the dwell time was considered at least 30 s for all the welds in order to enhance interface temperature. The rest of the input parameters for both the FSW as well as the TIG processes are given in Table 2. The novel technique of using an external water-cooling system through a pipe was employed for cooling-assisted friction stir welding (CFSW), as shown in Figure 1. The external water-cooling, flowing at a rate of 120 mL per minute, was used simultaneously with the exerting plunge step. Also, the argon shielding gas was set to about 5 L/min, thus impeding surface oxidation at the welding zone.

**Figure 2:**
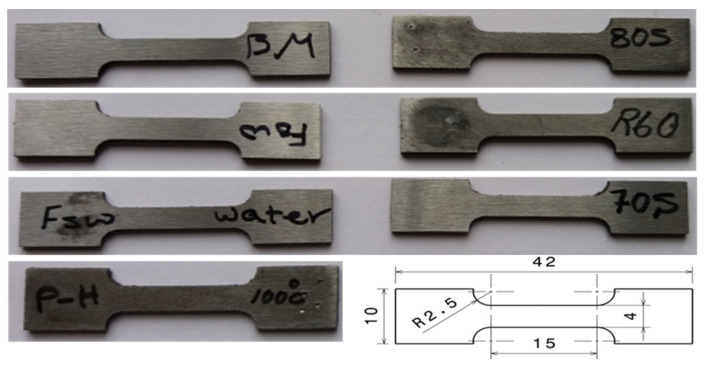
Scheme of tensile specimens welded through both FSW and TIG.

The samples from transverse cross-section of joints were prepared for microstructural investigations using the standard metallographic practice comprising mechanical grinding and polishing. The mounted samples were mechanically ground up to 5000 grit and finally polished with 1 μm diamond paste. To reveal the microstructural features, the specimens were etched with 2% Nital (98 mL ethanol + 2 mL nitric acid) reagent. The samples were examined for microstructural features in a light optical microscope (LOM). According to the recommendations of ASTM E 384-05a standard [17], Vickers microhardness measurements of the welds were made at a depth of 1.5 mm from the upper surface along the transverse cross-section of the samples by applying a load of 200 g for 12 s. The tensile properties of longitudinal specimens, prepared as per JIS Z2201 (type VII) guidelines (see Figure 2), were determined by testing in a AG-25GB Shimadzu machine (Shimadzu Co., Kyoto, Japan). Uniaxial tests were conducted at a crosshead speed of 1 mm/min at room temperature.

## 3. Results

### 3.1. Macro- and Microscopic Analysis

**Figure 3:**
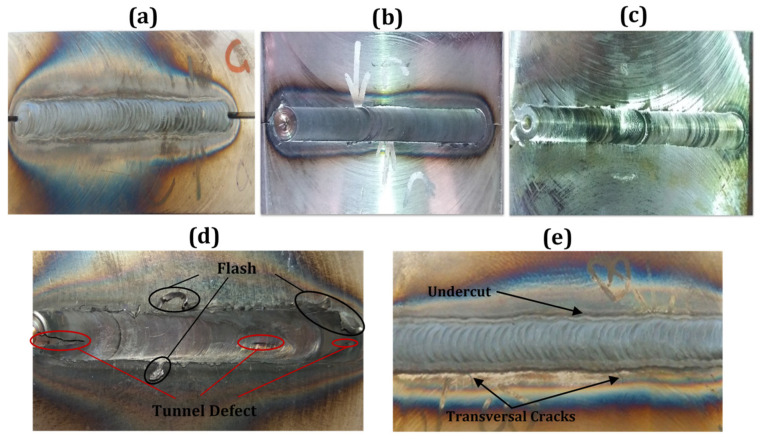
Visual inspection of the surfaces of the welds carried out under various process conditions: (**a**) TIG welded sample with ER70S-6 filler metal, (**b**) friction stir welded (FSWed) sample (950 rpm, 24 mm/min), (**c**) cooling-assisted friction stir welded (CFSW) sample (950 rpm, 24 mm/min), (**d**) FSWed sample (950 rpm, 32 mm/min), (**e**) TIG welded sample (150 A, 1 mm/s).

CK45 carbon steel plates were successfully welded using TIG, FSW and cooling-assisted friction stir welding (CFSW) techniques and the surface morphologies of defect-free joints are given in Figure 3a–c, respectively. The filler material was deposited on the top side of the TIG joint along the weld line. Visual observations of FSW and TIG welded specimens revealed that a careful selection of process parameters could enable realization of defect-free joints. In this context, examinations of preliminary FSW trials revealed weld defects, such as tunnel-type defect and flash at traverse speeds higher than 32 mm/min as shown in Figure 3d. The tunnel-type defect formed on the surface of the welds due to low heat input and insufficient material flow. These results are in agreement with the weld features reported on a SAF 2205 duplex stainless steel by Saeid et al. [18] that evidenced the achievement of defect-free joints at relatively low welding speed.

In Figure 3e, a picture of the TIG welded sample with undercutting and crack defects is shown. TIG welding had a high inclination towards forming undercutting defect by augmenting the travel speed. Therefore, based on studies concerning the occurrence of undercutting defects, levying an upper limitation on travel speed could be beneficial for conventional TIG welding [19]. At low welding currents (below 100 A), undercutting defects formed at high traverse speeds. In contrast, at high welding currents (above 150 A), low traverse speed led to the formation of undercutting defects, thus indicating the need for optimizing the welding current as well as traverse speed to mitigate formation of these defects. Another weld defect seen in the TIG welded sample was the occurrence of transversal cracks. These cracks started from the weld zone and spread to the base material. Extreme heat conditions went through filler metals and adjacent material causing a substantial thermal expansion of the surrounding material of the weld zone. Since the base material was still near room temperature, the dimensional inconformity brought about large residual tensile stresses resulting in transversal cracks. Thus, sound joints are achievable for both FSW and conventional TIG welding methods, only by setting optimized input parameters.

**Figure 4:**
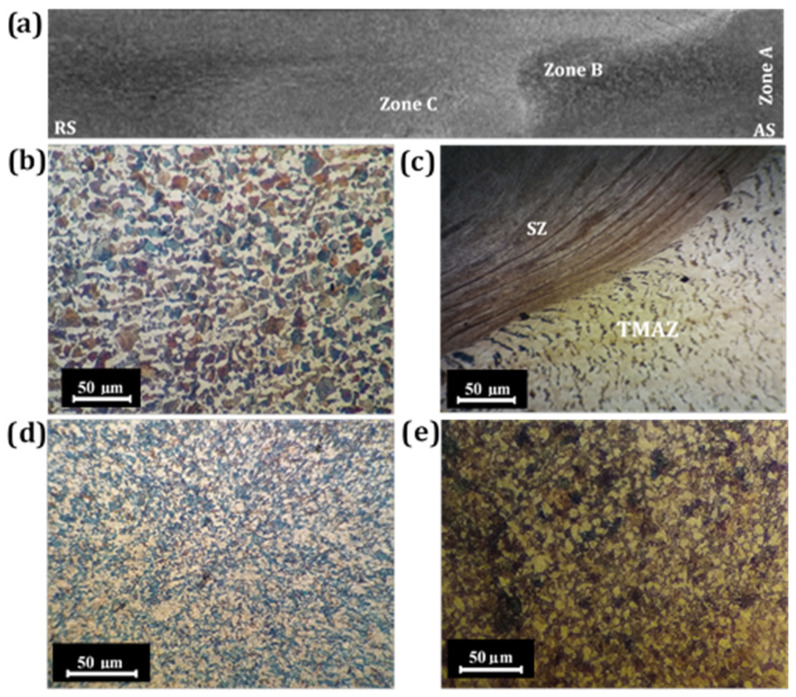
Macroscopic and microscopic evaluation of different zones in FSWed specimens (950 rpm, 24 mm/min): (**a**) macrograph depicting cross sectional plane perpendicular to FS weld direction; and microstructures of (**b**) base material, (**c**) stir zone/thermo-mechanically affected zone (SZ/TMAZ) border in FSW (**d**) Zone C in FSW and (**e**) CFSW.

Low-magnification images of the welds taken on cross-sectional plane perpendicular to the weld direction are depicted in Figure 4 for the friction stir welded (FSWed) CK45 steel sample. Regarding the transverse cross-sectional plane of FSWed plates, both the right and left sides of the weld zone center are pertaining to the advancing (AS) and retreating (RS) sides of the rotational tool, respectively. On the AS, the interface line between stir zone (SZ) and the thermo-mechanically affected zone (TMAZ) was recognizable, while diffusion at RS was better. This is ascribed to the asymmetry of the FSW process [20]. Optical micrographs of zones A, B and C presented in Figure 4a correspond to the base material, boundary of the TMAZ and SZ, and weld center, respectively. The microstructure of Zone A corresponds to the BM following normalizing of the steel sample studied in this experiment (see Figure 4b). The BM has a structure consisting of approximately 43% ferrite (α) matrix and 57% pearlite islands, which were estimated through quantitative metallography. The average grain size was about 49 µm. Zone B lies on the border of the SZ and TMAZ, which illustrates good continuity and defect-free weld (see Figure 4c). The SZ has taken the form of an oval-shaped nugget with an onion ring pattern. However, it is apparent that the heat-affected zone (HAZ) was not seen distinctly. The maximal temperature in the FSW of carbon steels surpasses 1100 °C using polycrystalline cubic boron nitride (PCBN) and tungsten-based tools. However, it has inevitably relied upon process parameters, welded materials, tool geometry and tool material [12]. Since the maximal temperature at the time of FSW process was above the Acm temperature, (i.e., about 850 °C for CK45 carbon steel in the present study), austenite grains must have grown, although the prior austenite grain boundaries could not be revealed clearly following nital etching, as shown in Figure 4d. The high peak temperature experienced in FS welding promoted phase transformation of austenite to martensite during the final cooling step. Therefore, about 49% martensite structure was produced during FSW in zone C resulting in enhanced micro-hardness as well as yield and tensile strengths (see Section 3.2.2). The microstructure of SZ obtained via the CFSW technique comprised of a ferrite matrix with 41% pearlite islands (see Figure 4e). Although the microstructure of CFS welded plates resembled BM, the SZ of CFSW contained finer pearlite and ferrite grains, with an approximate grain size averaging 15 µm. This result signified that the water-cooling system in CFSW led to the lower peak temperature unlike in the case of air-cooled FSW plates, thus resulting in the formation of relatively homogeneous and finer austenite grains in the SZ. Therefore, the relatively uniform and fine microstructure comprising ferrite and pearlite phase constituents obtained through continued water cooling during CFSW ought to result in improved weld quality and properties.

**Figure 5:**
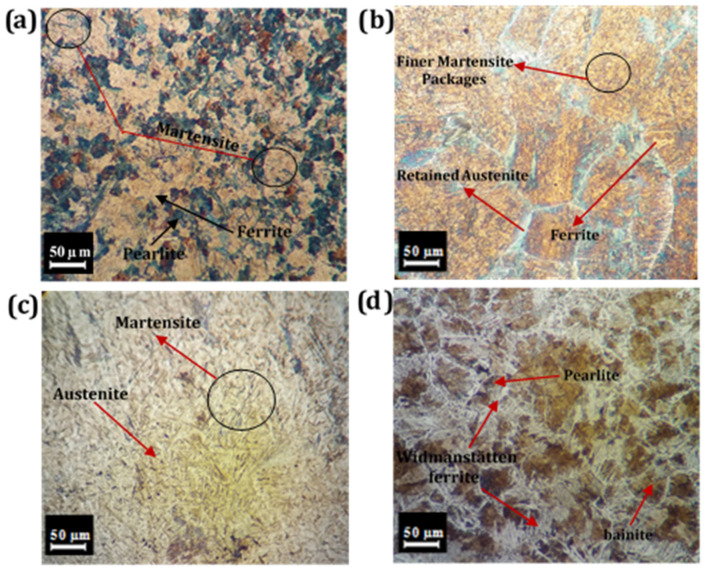
Microstructures of TIG welded samples using different filler metals: (**a**) without filler metal, (**b**) ER70S-6 filler metal, (**c**) ER80S-B2 filler metal and (**d**) R60 filler metal.

The microstructural observations of the nugget zone (NZ) in TIG welded plates, with and without the use of filler metals, are presented in Figure 5. It is well known that the mechanical and microstructural properties of TIG welded samples are considerably affected by the heat flow direction, cooling rate and the chemical composition of filler metals [21]. It could be discerned from Figure 5a that the microstructure of the TIG welded samples without using any filler metal consisted primarily of ferrite and pearlite, resembling the BM. However, TIG welding of the sample with ER70S-6 filler metal imparted formation of mainly fine martensite packets in NZ with small pockets of ferrite and retained austenite, as shown in Figure 5b. This microstructure could have formed through a cooling cycle of the (ferrite + austenite) microstructure, leading to the phase transformation of austenite to martensite. Similarly, as with the TIG welded sample using ER70S-6 filler metal, the ER80S-B2 filler metal also developed in principle a martensitic microstructure, owing to the presence of Mo in the filler, thus resulting in improved hardenability of the NZ. Presence of Mo in both the BM as well as filler metals (ER70S-6 and ER80S-B2) led to the martensitic transformation in NZ because of the improved hardenability of the welded joint, thereby reducing the weldability of samples substantially. However, TIG welded sample with ER80S-B2 filler metal had a relatively higher amount of Mo, resulting essentially in a high fraction of martensite with only a very small fraction of austenite (see Figure 5c). Figure 5d shows the NZ of sample TIG welded using R60 filler metal that comprised of bainite, Widmanstätten ferrite (WF) and some area fraction of pearlite. The presence of Widmanstätten ferrite and bainite phases in the NZ signified that the temperature of this region exceeded Acm. Both the Widmanstätten ferrite and bainite are transformed from austenite with discrete orientation. 

### 3.2. Mechanical Properties

#### 3.2.1. Hardness

**Figure 6:**
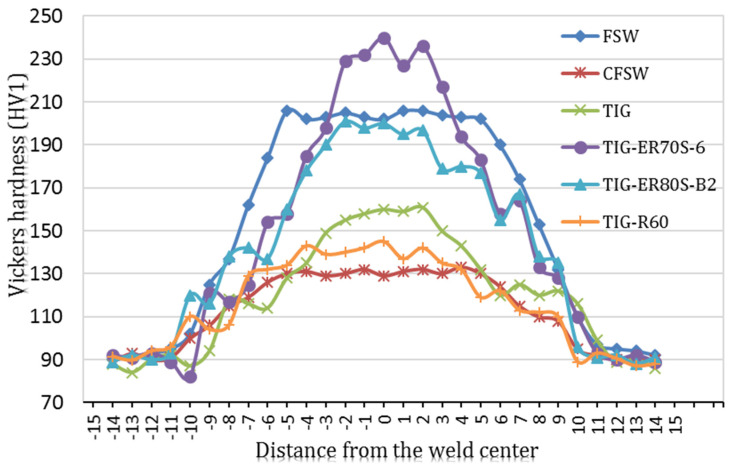
Hardness profiles for both FSW and TIG joints.

Figure 6 exhibits the Vickers microhardness profiles of the mid-section of the CK45 carbon steel weldments after employing both the FSW and TIG welding processes. It is to be noted that the average hardness of the BM was 93 ± 3 HV. In general, by proceeding from the base material toward the fusion and stir zones, the microhardness went through an incremental trend depending on various welding conditions. This result could be attributed to the martensite transformation obtained via rapid cooling rates. However, the hardness profile of CFSWed samples was not significantly affected compared to what was reported in the stir zone of FSWed welds, obviously as a result of lower peak temperature in the case of water-cooling assisted methods. Comparing the microhardness profiles of both the TIG and FSW weldments, it can be outlined that TIG welded samples have a higher reduction in hardness in the weld line than those of FSW joints owing to a higher heat input into the weld zone. Microhardness results revealed hard and brittle weld zones for both welding methods, particularly for typical FSW specimens as well as TIG welded samples with ER70S-6 filler metal. With the higher hardness obtained in the weld zones in comparison to the base metals, the corresponding microhardness results of all weldments are in good agreement with the tensile outcomes.

#### 3.2.2. Tensile Properties

**Figure 7:**
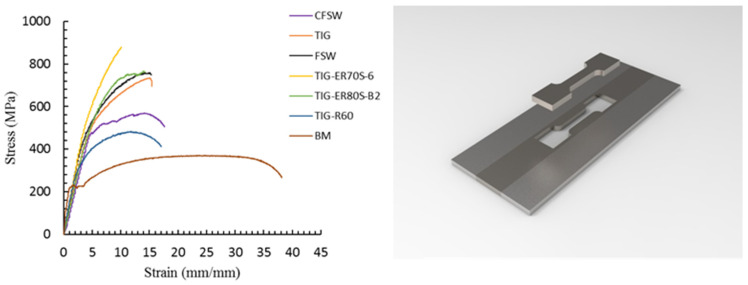
Tensile stress-strain curves of FSWed and TIG welded samples.

Figure 7 depicts the engineering stress-strain curves plotted for BM, FS welded and TIG welded samples. It is worth mentioning that the tensile properties were measured on longitudinal samples taken from the stir zone and the results are shown in Table 3. As can be seen from the table, both the yield and tensile strengths nearly doubled in FS welded sample. Since the peak temperature of the SZ during FSW reached almost 0.6–0.9 times the melting point of weld materials and the cooling process occurred in air soon after the welding, significant formation of martensite phase occurred causing considerable increment in tensile strength and a consequent decrement in elongation (27%). The results are in agreement with those reported by Haghshenas and Gerlich [22]. However, the tensile strength of the CFSW sample decreased to 570 MPa, while elongation experienced a 3% augmentation because of the lower heat input and a higher cooling rate, leading to the formation of fine pearlite and ferrite grains in the microstructure. In the case of TIG welded samples, tensile strengths ranged from 483 MPa to 878 MPa depending on the filler materials. Although both tensile strength and yield strength were affected as a consequence of welding operations, filler materials played a pivotal role in the tensile properties of weldments. The TIG welded sample using ER70S-6 filler metal recorded a maximum increase in tensile strength and the lowest elongation, while the specimen with R60 filler metal possessed better ductility in comparison to the other TIG weldments. It can be noticed from the table that the optimal strength and ductility in relation to the BM are attained via both CFSW and TIG welding process as using R60 filler metal. This may be related to the merits of ferrite-based structure achieved in both welding methods used for CK45 carbon steel.

The results obtained in respect of hardness data and tensile properties using both the friction stir welding as well as fusion welding methods revealed that sound joints with good mechanical properties would be attainable, if the process parameters are carefully selected, with an appropriate choice of filler metal in the case of TIG welding procedure.

## 4. Conclusions

An empirical study was performed on the evaluation of microstructures and mechanical properties obtained on CK45 carbon steel welded via FSW and TIG welding methods. Based on the results achieved, the following concluding remarks can be made:

- Sound joints could successfully be accomplished via CFSW procedure with the following parameters: rotational speed 950 (rpm), traverse speed 24 (mm/min), plunge depth 0.4 (mm), and tilt angle 0.3 (degree);
- The optimal mechanical property combination was accomplished in CFSWed plates, wherein the microstructure resembled BM. However, the SZ of CFSWed plates contained a finer ferrite-pearlite microstructure, owing to the lower heat input generated, in comparison to typical FSW;
- In TIG joints, the nugget zone (NZ) of welded samples with ER70S-6 and ER80S-B2 filler metals possessed essentially a martensite structure that was attributed to the presence of Mo in both BM and filler metals, which not only increased the hardenability of the materials, but also reduced the weldability of plates dramatically. In contrast, the NZ of a TIG welded sample using R60 filler metal consisted of a mix of bainite and Widmanstätten ferrite (WF) structure, thereby imparting reasonable mechanical properties;
- From the industrial application point of view, friction stir welding process stays highly competitive in this fast-changing world embracing new technologies due to both energy-saving as well as preventing the joints from getting riddled with defects, which are often seen in the case of fusion welding methods.

## Figures and Tables

**Table 1:** Nominal chemical compositions of CK45 carbon steel plates and filler metals.

Type	Chemical Composition (%)									
	Fe	C	Si	P	S	Cr	Mo	Ni	Mn	Cr, Mo, Ni
CK45	balance	0.42–0.5	0.19	≤0.3	≤0.035	≤0.4	≤0.1	≤0.4	0.5–0.8	≤0.63
R60	balance	0.1	0.17	0.013	0.016	0.07	0.02	0.1	0.93	-
ER70S-6	balance	0.08	0.8	0.014	0.03	0.025	0.4	0.013	1.44	-
ER80S-B2	balance	0.1	0.4	0.02	0.02	1.2	0.4	0.2	0.4	-

**Table 2:** Welding input parameters.

Welding Conditions			
Welding Type	Process Parameters	Unit	Values
TIG	Welding current	(A)	120 ± 10
	Arc length	(mm)	2
	Welding speed	(mm/s)	1.1
	Voltage	(V)	12–12.8
	Shielding gas	-	Argon
	Filler material	-	R60, ER70S-6, ER80S-B2
FSW	Rotational speed	(rpm)	950
	Traverse speed	(mm/min)	24
	Plunge depth	(mm)	0.4
	Tilt angle	(degree)	3

**Table 3:** Mechanical properties of base metal (BM), FSWed and TIG welded samples.

Sample	Yield Strength (MPa)	Ultimate Tensile Strength (MPa)	Total Elongation (%)
Base material	224 ± 1	381 ± 2	37 ± 2
FSW	459 ± 3	757 ± 5	10 ± 1
CFSW	477 ± 3	570 ± 4	13 ± 1
TIG without filler metal	395 ± 2	551 ± 4	11 ± 1
TIG with ER70S-6 filler metal	551 ± 5	878 ± 7	4 ± 0.3
TIG with ER80S-B2 filler metal	488 ± 4	766 ± 6	8 ± 0.6
TIG with R60 filler metal	311 ± 3	483 ± 5	14 ± 1

## Data Availability

Data is contained within the article.
